# Association of Intra-articular Ozone/Prolozone Injections With Cutibacterium acnes Infection in a Shoulder With Pseudogout and No Prior Surgery: Management Principles and Literature Review

**DOI:** 10.7759/cureus.112479

**Published:** 2026-07-11

**Authors:** Brett W Richards, Kevin B Curtis, Joseph A Richards, Amishi Rohaj, Joel D Trachtenberg, Pankhuri Gupta, John G Skedros

**Affiliations:** 1 Shoulder and Elbow, Utah Orthopaedic Specialists, Salt Lake City, USA; 2 Infectious Disease, Intermountain Healthcare, Salt Lake City, USA; 3 Rheumatology, Intermountain Healthcare, Salt Lake City, USA

**Keywords:** corticosteroid injection, osteoarthritis, ozone therapy, pseudogout, septic arthritis, shoulder joint

## Abstract

Septic arthritis following joint injection/aspiration is an uncommon and challenging orthopaedic disease. We report a unique case of a 58-year-old non-diabetic, non-obese female with no prior shoulder surgery who developed septic arthritis of the right shoulder due to the anaerobic bacterium *Cutibacterium acnes *(*C. acnes*), identified from deep-tissue samples obtained during arthroscopic debridement. The case is notable for a history of pseudogout and multiple recent intra-articular injections in the affected shoulder. The patient received four shoulder joint (glenohumeral) ozone/prolozone injections over a one-month period for shoulder pain attributed to pseudogout with glenohumeral osteoarthritis. Within one week of the final ozone/prolozone injection, the pain markedly worsened, and a large subdeltoid/glenohumeral effusion was detected grossly and via MR imaging. The subdeltoid effusion was confluent with the glenohumeral joint through a nontraumatic rotator cuff tear. Initial management included aspiration of the effusion (55 mL) and corticosteroid injection. This was followed by three additional aspirations over the next three months, one of which also included another corticosteroid injection. Hence, there were eight independent percutaneous needle punctures into the shoulder joint/effusion over six months. The tissue cultures that grew *C. acnes* were obtained five weeks after the final (eighth) aspiration/injection. This report highlights five key considerations and issues of this highly unusual case: (1) the potential causal association between repeated intra-articular injections/aspirations and subsequent septic arthritis with *C. acnes*; (2) the rare occurrence of septic arthritis following intra-articular ozone/prolozone injections; (3) the association with pseudogout and the later-postulated diagnosis of seronegative rheumatoid arthritis, (4) the potential value of enhanced skin preparation protocols in reducing *C. acnes *infection risk; and (5) the challenge of attributing causality, given that *C. acnes *can exist as a native joint commensal organism or as a culture contaminant, with another organism being the true pathogen (e.g., coagulase-negative Staphylococci).

## Introduction

Anaerobic bacterial infections of large joints, such as the shoulder, knee, and hip, are rare in the absence of prior surgical intervention or implanted hardware. The anaerobic bacterial species *Cutibacterium acnes* (*C. acnes*, formerly known as *Propionibacterium acnes*) is the most common cause of postoperative infections of the shoulder (glenohumeral joint) [1]. When anaerobic or aerobic bacterial infections occur in the shoulder or other joints without prior surgery, this is referred to as native joint septic arthritis [2,3]. In contrast, when joint sepsis occurs following a therapeutic injection (e.g., with a corticosteroid), we propose the term procedure-associated septic arthritis (PASA) to describe this condition. PASA is not an established term in the literature but is introduced here to clarify that this condition is not a *de novo* infection of the native joint, but rather an iatrogenic infection associated with a non-surgical “procedure” typically performed in a clinic (such as the case described herein).

While PASA of the shoulder joint is rare (details of our literature search are presented below), it is even more unusual following ozone-based therapeutic intra-articular injections. This is because, as described below, ozone is hypothesized to be protective against infection. We report the unusual case of a middle-aged female who developed an anaerobic bacterial infection of her right shoulder joint with *C. acnes* after a series of eight therapeutic injections, including both ozone/prolozone (defined below) and corticosteroids for shoulder pain. This case illustrates the risk of infection from repeated percutaneous joint access and the lack of standardized practice surrounding non-traditional therapies. To best understand this case, it is important to distinguish between three related but distinct injection-based treatments that are often conflated in the literature, as discussed below.

Ozone therapy

When used to treat musculoskeletal disorders, ozone therapy involves the injection of a medical-grade oxygen-ozone (O2-O3) gas mixture into intra-articular, periarticular, intramuscular, or other locations [4-6]. Proposed mechanisms include analgesic, anti-inflammatory, and antioxidant effects [4,7], and ozone is hypothesized to be protective against infection [8,9]. However, this antimicrobial effect is not fully established (see Discussion section).

Prolotherapy

Prolotherapy (proliferative therapy) is a complementary and alternative medicine technique used to treat musculoskeletal pain [10-12]. It involves injecting an irritant solution, often hyperosmolar dextrose, into joints or near ligaments and tendons. It is administered as a liquid and does not include gas. Unlike corticosteroids, which suppress inflammation, prolotherapy is hypothesized to trigger a localized inflammatory response by stimulating the release of growth factors or acting as a vascular sclerosant [12,13].

Prolozone therapy

Prolozone therapy, a contraction of “proliferation” and “ozone,” is a non-standardized hybrid technique combining ozone/oxygen gas with other agents (e.g., anesthetics, anti-inflammatory medication, homeopathics, vitamins, minerals, proliferatives, and/or corticosteroids) [14-18]. In contrast to traditional corticosteroid injections, which can weaken tissues with repeated use [19-21], prolozone therapy is intended as a regenerative therapy, though this is not proven.

## Case presentation

**Figure 1:**
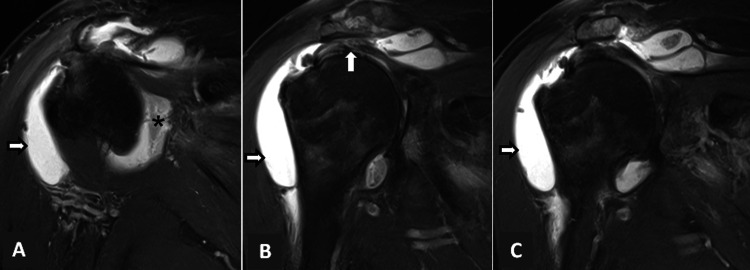
MRI of our patient's right shoulder Magnetic resonance (MR) images taken in the coronal plane at the anterior (A), middle (B), and posterior (C) aspects of the patient’s right shoulder joint. No contrast agent was used; hence, the fluid indicated by the whiter areas represents the effusion that fills the subdeltoid/subacromial region (left side arrows) and is confluent with the glenohumeral joint (asterisk in A) through a rotator cuff tendon tear (vertical arrow in B). The images are 10 mm apart.

Our patient is a 58-year-old, right-hand-dominant Caucasian female from the United States (height: 167.6 cm; weight: 72.8 kg; BMI 25.9 kg/m^2^) with no history of obesity or diabetes. She presented to our clinic in late March 2025 with chronic right shoulder pain and recurrent, grossly apparent subacromial effusions. Demographic characteristics are relevant given the reported variability in the incidence and antibiotic resistance of *C. acnes* across populations [1,22-24]. Her shoulder pain became so severe that it caused her to temporarily discontinue several activities of daily living, including her occupation as a hairstylist. She had a several-year history of pseudogout (calcium pyrophosphate (CPP) deposition disease) and moderate glenohumeral osteoarthritis of the right shoulder without prior shoulder surgery. MRI scanning (without a contrast agent or skin puncture) also revealed a degenerative full-thickness rotator cuff tear (Figure 1).

She denied tobacco or illicit drug use, recent dental work, and other potential sources of local or hematogenous seeding. She also lacked conditions known to be associated with *C. acnes* infection, including sarcoidosis, synovitis, acne, pustulosis, hyperostosis, osteitis (SAPHO) syndrome, and chronic recurrent multifocal osteomyelitis [1]. She had benign essential hypertension treated with amlodipine and hydrochlorothiazide, depression treated with duloxetine, and chronic joint pain (primarily shoulders and knees) managed with methotrexate and occasional gabapentin. Two prior rheumatoid factor tests and an antinuclear antibody (ANA) test were negative. Throughout the clinical course described in this report, she denied fevers, chills, or sweats, as well as neurological symptoms. Furthermore, she had no significant history or evidence of acne.

**Figure 2:**
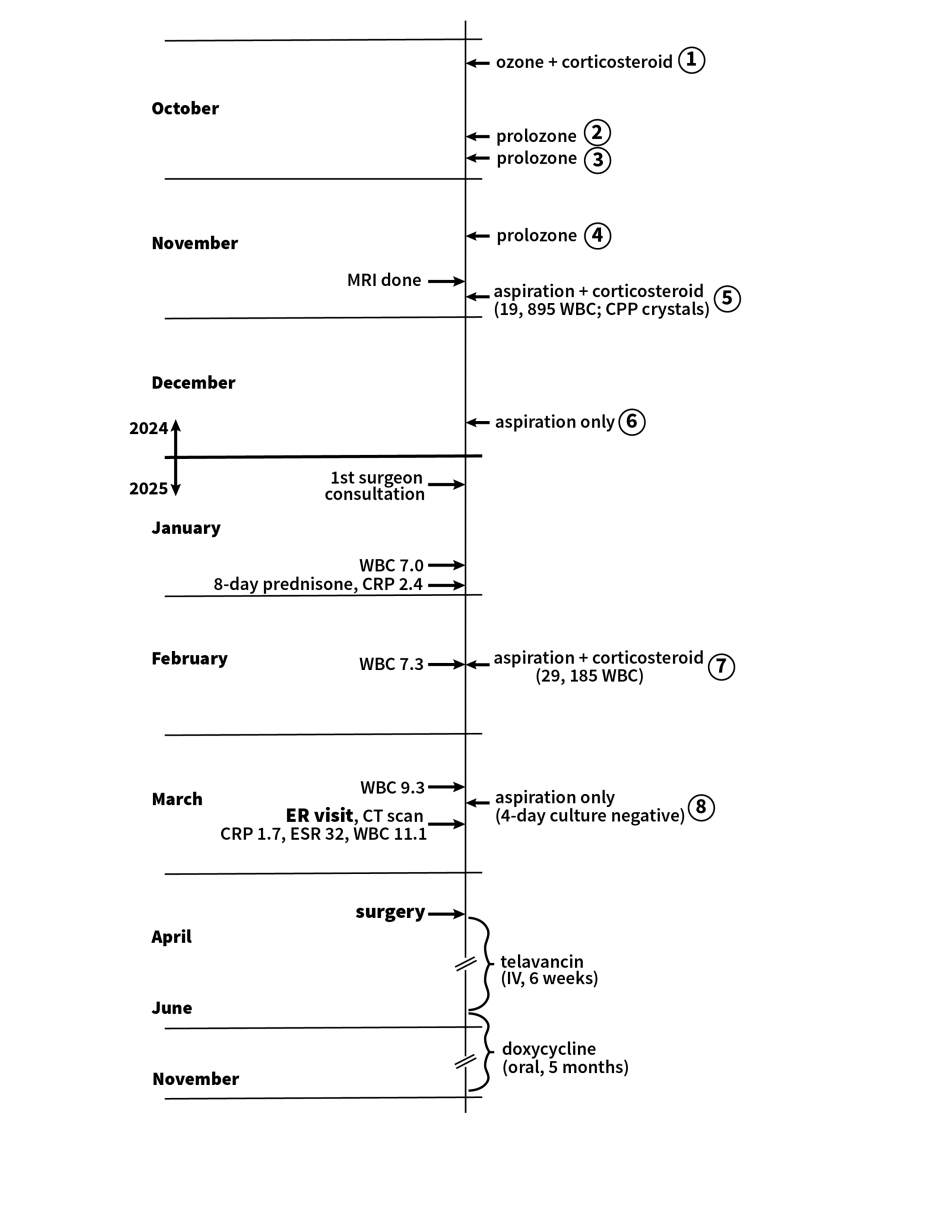
Timeline of injections and clinical events This image was digitally created for the purpose of this publication using Adobe Illustrator (credit, Mark T. Nielsen, Professor Emeritus of Anatomy, University of Utah). CPP = calcium pyrophosphate CRP = C-reactive protein

**Figure 3:**
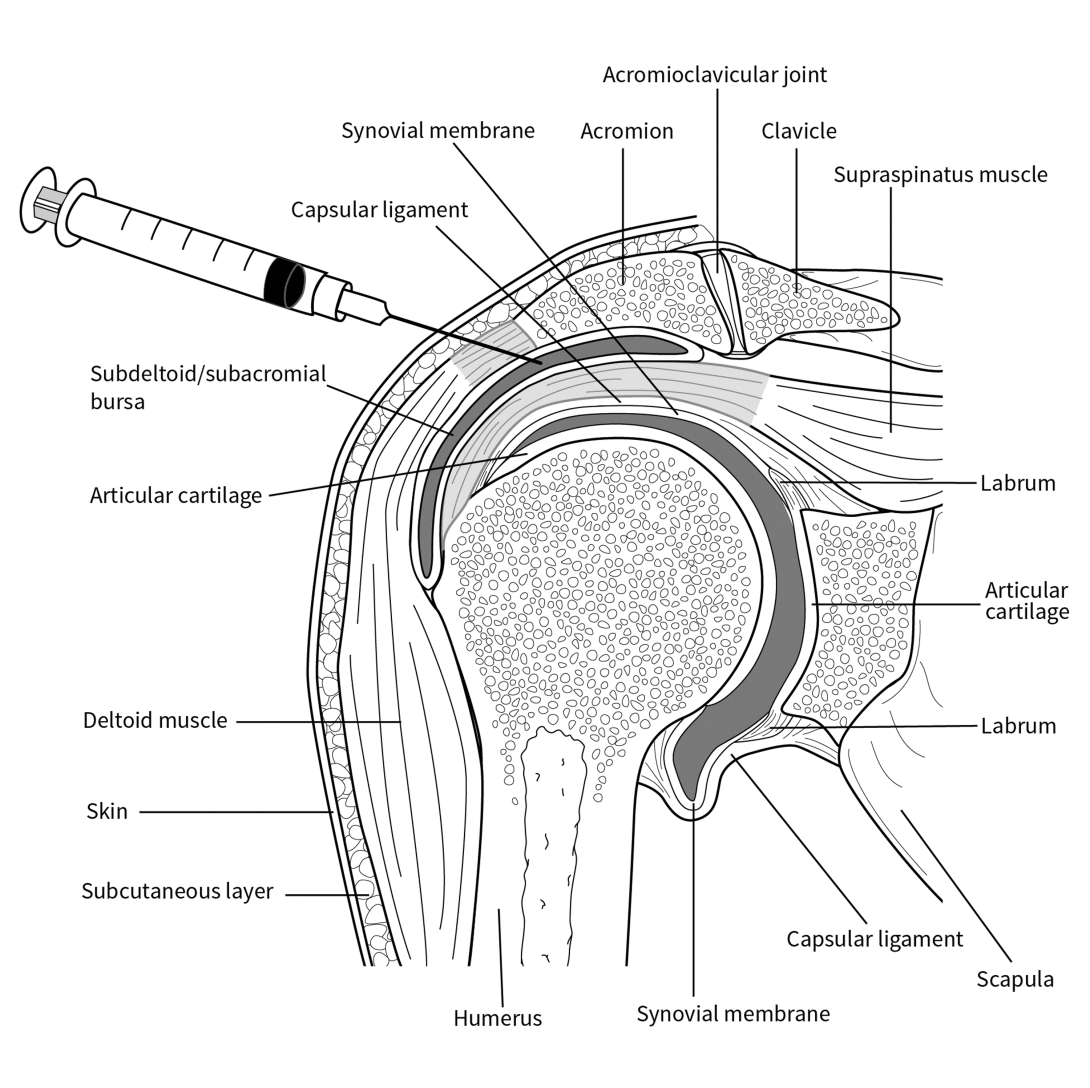
Drawing of normal shoulder with a needle in the subacromial space Drawing of normal shoulder with a needle in the subacromial space (i.e., within the subacromial bursa) where injections for shoulder impingement syndrome are typically given. Note that the subdeltoid/subacromial bursa and the glenohumeral joint are separate spaces. This image was digitally created for the purpose of this publication using Adobe Illustrator (credit, Mark T. Nielson, Professor Emeritus of Anatomy, University of Utah).

Between October and November 2024 (five to six months before presenting to our clinic), the patient received a series of four intra-articular ozone/prolozone injections (Figure 2) for pain and inflammation in her right shoulder (glenohumeral) joint (Figure 3). The injections were done (without ultrasound guidance) on four separate days by the same provider at a wellness facility specializing in homeopathic/alternative treatments. Details regarding these procedures were obtained through review of outside medical records. However, the records did not specify the skin antisepsis protocol used before injection. Additional attempts to clarify procedural details, including the specific components of the proliferative/additive mixture used in injections two to four, the skin preparation used, and the type of ozone generator used, were unsuccessful.

These first four injections consisted of the following: (1) ozone + corticosteroid injection in early October 2024: 8 ml (33 gamma (µg/ml)) ozone and 40 mg triamcinolone acetonide (injectable suspension, USP) with 2% lidocaine (this is a standard corticosteroid dose for a shoulder joint injection for osteoarthritis [25]); (2) prolozone injection in late October 2024: 8 ml ozone (33 gamma) and 2.5 ml of a mixture of proliferatives and other additives; (3) prolozone injection four days after the second injection: 8 ml ozone (33 gamma) and 2 ml of the proliferative/additive mixture; and (4) prolozone injection in early November 2024: 8 ml ozone (33 gamma) and 2 ml of the proliferative/additive mixture.

**Figure 4:**
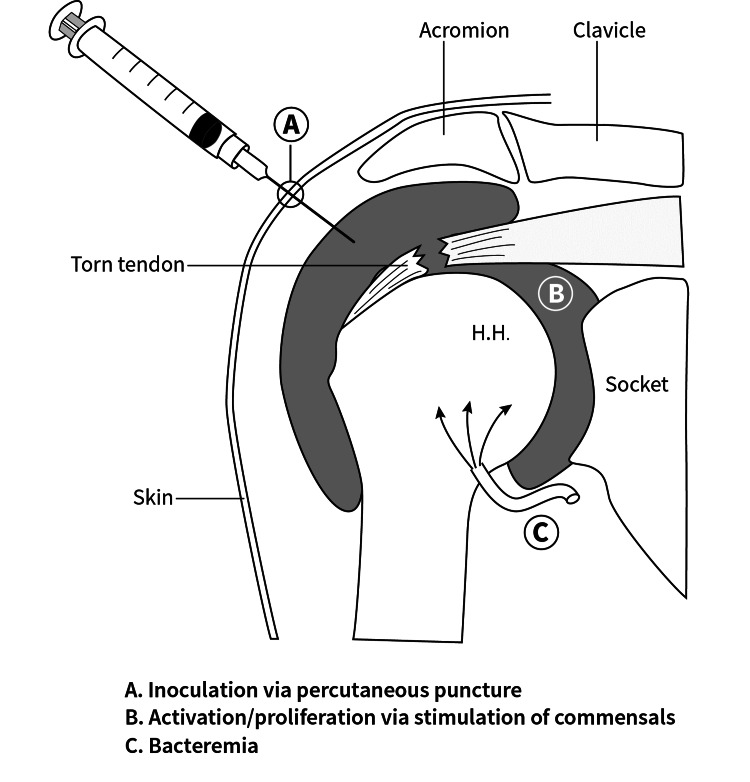
Diagram of our patient's shoulder with torn rotator cuff and large effusion Diagrammatic drawing of our patient’s shoulder in accordance with the MR images shown in Figure 1. Potential routes of infection are shown as A, B, and C, and are intended as illustrative hypotheses rather than findings directly supported by clinical evidence. The needle at left is in the subdeltoid/subacromial effusion (shown in gray). As shown, the effusion extends from that region and into the glenohumeral joint (also gray) through a rotator cuff tendon tear (vertical arrow in Figure 1B). This image was digitally created for the purpose of this publication using Adobe Illustrator (credit, Mark T. Nielsen, Professor Emeritus of Anatomy, University of Utah). H.H. = humeral head

Approximately five days after the final prolozone injection, the patient experienced worsening right shoulder pain and swelling and sought consultation with an orthopaedic shoulder surgeon unaffiliated with our clinic. This resulted in referral to a non-surgeon physician for an ultrasound-guided aspiration. In late November 2024, that provider aspirated 55 mL of mildly cloudy serosanguineous fluid (Figure 4) and injected a corticosteroid (40 mg triamcinolone acetate) through the same needle. Fluid analysis showed CPP crystals and a white blood cell (WBC) count of 19,875 cells/µL; notably, no cultures were sent at that time, the first of several early diagnostic limitations in this case (the only cultures that were sent were in mid-March 2025, as described below). A summary of the laboratory results obtained throughout the course of treatment is provided in Table 1. She was then treated with colchicine and oral nonsteroidal anti-inflammatory drugs (NSAIDs; ketorolac tromethamine followed by meloxicam) for pain attributed to subacromial impingement syndrome associated with glenohumeral pseudogout and osteoarthritis.

**Table 1:** Summary of laboratory results obtained throughout the course of treatment CRP = C-reactive protein (normal < 0.5 mg/dL) ESR = erythrocyte sedimentation rate (normal < 30 mm/hr) WBC = white blood cell count (normal 3,600 - 10,600 cells/mL) CPP = calcium pyrophosphate *  = abnormal value -  = not measured

LAB RESULTS									
A. Blood Tests and Right Shoulder Aspirates									
Date	WBC		CRP (mg/dL)		ESR (mm/hr)		Shoulder Aspirate Volume (ml)	Shoulder Aspirate WBC (cells/µL)	Shoulder Aspirate Crystals
Nov. 2024	-		-		-		55	19,875	CPP
Dec. 2024	-		-		-		45	-	CPP
Jan. 2025	7,000		2.4*		-		-	-	-
Feb. 2025	7,300		-		-		45	29,185	-
Early Mar. 2025	9,300		-		-		-	-	-
Mid-Mar. 2025	-		-		-		30	-	-
Late Mar. 2025	11,100*		1.7*		32*		-	-	-
B. Left Knee Aspirates									
Date		Knee WBC		Knee Crystals	
May 2025, Aug. 2025, Nov. 2025		3,000-15,000		None	

The timeline of the final series of four shoulder injections/aspirations and additional blood tests is summarized in Figure 2. These final four shoulder injections/aspirations were done with ultrasound guidance by the same provider, with standard skin preparation (2% chlorhexidine gluconate/70% isopropyl alcohol (ChloraPrep; Becton Dickinson, Franklin Lakes, NJ, USA)) before each procedure. Subsequent computed tomography (CT) scanning, without joint contrast fluid or skin puncture, confirmed the prior MR findings of a large subdeltoid/subacromial effusion confluent with the glenohumeral joint through a full-thickness tear of the supraspinatus tendon (Figures 1, 4).

In mid-December 2024, repeat aspiration of the recurrent effusion yielded 45 mL of mildly cloudy yellow fluid. Analysis again revealed CPP crystals. Cell counts and cultures were not done, and no corticosteroid was injected. Because the effusion recurred four days later and the pain did not improve significantly, she was switched to diclofenac and tramadol. Arthroscopic surgery (subacromial bursectomy/decompression/debridement) was considered but deferred, as the consulting surgeon felt it was unlikely to outperform optimal medical management of her pseudogout. Although septic arthritis was included in the differential diagnosis, it was considered low probability. Given the severity of her glenohumeral arthritis, she was also informed that total shoulder arthroplasty was a future possibility.

In late January 2025, the patient’s rheumatologist found normal rheumatoid factor, thyroid function, and WBC, but elevated C-reactive protein (CRP) at 2.4 mg/dL (normal <0.5 mg/dL). She was prescribed an eight-day oral prednisone taper (80 to 20 mg) for continued joint pain. Subsequent testing for cyclic citrullinated peptide (anti-CCP) antibodies was negative, suggesting either a low probability of rheumatoid arthritis (RA) or, alternatively, seronegative RA or a less aggressive disease phenotype [26,27].

In mid-February 2025, the patient underwent another subacromial aspiration (45 mL, serosanguineous) followed immediately by a corticosteroid injection (40 mg triamcinolone acetate) through the same needle. Fluid analysis showed 29,185 WBCs/µL; cultures were not sent. In mid-March 2025, a final subacromial aspiration (30 mL, serosanguineous) was sent for culture (no corticosteroid was injected), but no growth was detected. The culture was held for only four days, well short of the 14 days recommended for detecting the slow-growing *C. acnes* (discussed below).

In late March 2025, the patient presented to our hospital’s emergency department with increasing shoulder pain and swelling, without fevers, chills, or sweats. Blood tests revealed mild elevations in CRP (1.7 mg/dL, normal < 0.5), erythrocyte sedimentation rate (ESR; 32 mm/hr, normal < 30), and WBC (11,100 cells/mL, normal 3,600 - 10,600). Notably, this was the only elevation in WBC among four blood samples obtained from January to April 2025 (Figure 2). A comprehensive metabolic panel, including electrolytes, glucose, blood urea nitrogen, and creatinine, was otherwise within normal limits. No aspiration or urgent intervention was recommended at that time, and she was referred to our shoulder specialty clinic. Given her history of recurrent effusions and eight prior shoulder injections/aspirations within the prior six months, surgical debridement with collection of multiple sterile tissue specimens was recommended to rule out an anaerobic infection.

In early April 2025, the patient underwent arthroscopic debridement surgery of the right shoulder with deep tissue biopsy, performed by J.G.S. Prophylactic antibiotics were withheld until all tissue samples had been collected. Notably, the patient had not taken any antibiotics during the preceding 12 months, including the time of the injections/aspirations described herein. At the start of arthroscopy, 20 mL of mildly cloudy yellow fluid was obtained and again showed CPP crystals. Gross observation of the shoulder joint showed marked inflammation, characterized by diffusely erythematous and thickened synovial lining. To the surgeon, who had 30 years of experience as a shoulder specialist, these observations appeared consistent with infection.

In accordance with published protocols, five synovial tissue samples were obtained from different sites within the inflamed joint using new instruments for each sample and submitted separately to reduce cross-contamination risk [28,29]. Samples were cultured for aerobic and anaerobic bacteria, acid-fast bacilli (AFB/mycobacteria), and fungi using standard Isolator and BACTEC systems (Wampole Laboratories, Cranbury, NJ, USA; Becton Dickinson Diagnostic Instruments Systems, Sparks, MD, USA). The anaerobic culture protocol included cooked meat glucose liquid broth, plating on anaerobic blood agar, and anaerobic blood agar containing phenyl ethyl alcohol (PEA) to select for Gram-positive organisms.

Two of the five tissue cultures grew *C. acnes* on solid medium, meeting the established threshold for true infection rather than contamination (defined as two or more of five positive cultures, whereas one of five is generally considered contamination) [28,30]. Growth was detected on day nine for each of the two positive cultures; the remaining three specimens were held for 14 days before being finalized as negative. *C. acnes* was identified by matrix-assisted laser desorption/ionization time-of-flight (MALDI-TOF) mass spectrometry. Phylotype/strain analysis was not done, though this information can be clinically important in select cases [1,31,32]. Fungal and acid-fast bacilli (e.g., *M. tuberculosis*) cultures were also negative.

Because *C. acnes* can be a skin contaminant, there was concern that this was actually a culture-negative arthritis caused by other slow-growing organisms such as biofilm-forming coagulase-negative staphylococci [33]. In view of this diagnostic uncertainty and for ease of outpatient antibiotic treatment, the patient received a six-week course of daily intravenous (IV) telavancin 750 mg (Figure 2), a broad-spectrum antibiotic effective against Gram-positive organisms [34] (penicillin is the first-line treatment for definitive cases of *C. acnes*). Unlike more standard antibiotics that only inhibit cell wall synthesis (e.g., vancomycin), telavancin also disrupts the bacterial membrane, leading to rapid bactericidal activity [35]. After completion of the IV antibiotic, oral doxycycline hyclate 100 mg twice daily was taken for five months (discontinued in November 2025) (Figure 2).

One month after surgery (mid-May 2025), the patient’s shoulder motion and pain had markedly improved. However, atraumatic left knee swelling with aching pain associated with recurrent effusions developed. The knee was aspirated on three separate occasions (May, August, and November 2025), each showing leukocytosis (WBC range: 3000-15000 cells/mL) and negative crystals and cultures, raising suspicion for seronegative RA. Because she was still completing antibiotic therapy, treatment for this presumptive diagnosis was with methotrexate.

In December 2025, daily low-dose prednisone (5 mg) was prescribed for persistent generalized joint pain. Because this treatment was inadequate for optimal symptom management, Hadlima (adalimumab-bwwd; Samsung Bioepis, Incheon, Korea), a biweekly injectable tumor necrosis factor (TNF) antagonist, was started. At 14 months after shoulder surgery, the patient had minimal shoulder pain, no evidence of recurrent infection, normalized inflammatory markers, and had not required additional surgery.

## Discussion

When this patient first presented to our clinic, several aspects of her case raised suspicion for infection: (1) a six-month history of right shoulder joint pain and recurrent effusions associated with eight prior intra-articular percutaneous needle punctures, and (2) concurrent pseudogout. Although our patient was later diagnosed with seronegative RA, which may have contributed to the development of her infection, management at the time was based on the assumption that her primary rheumatological disease was pseudogout. This is reflected in our discussion of potential contributing factors to our patient’s infection. There are myriad considerations raised by the presentation, differential diagnoses, and management of this case. Below, we address these considerations and outline various management principles applicable to cases of anaerobic bacterial PASA associated with prior therapeutic intra-articular ozone/prolozone and corticosteroid injections as well as *de novo* native joint septic arthritis (NJSA). Additionally, we conducted a literature review to identify reported cases of PASA and *de novo* NJSA in the shoulder attributed to *C. acnes*, as well as cases of large joint infection following ozone/prolozone injection. Factors that may have facilitated *C. acnes* infection in our patient are summarized in Table 2.

**Table 2:** Factors that may have increased our patient's risk of infection Potential factors at play in our patient in creating a joint environment favorable for biofilm-forming bacterial species like *C. acnes*. Factors are listed 1-7, with 1 being the most likely (evidence-based), and 5-7 being speculative/hypothesized possible mechanisms. Most factors are interrelated. CPP = calcium pyrophosphate, RA = rheumatoid arthritis

Factor	Mechanism
1. Needle punctures causing microtrauma/neovascularization	Repeated punctures introduce bacteria and disrupt tissue integrity; new vessel formation alters the local microenvironment and facilitates bacterial invasion [36-38].
2. Pseudogout crystals	CPP crystals potentially perturb the synovial membrane of synovial joints, creating a dysbiotic microenvironment that favors bacterial adhesion [37,39,40].
3. Seronegative RA	Chronic synovial inflammation and sub-synovial neovascularization may impair host defense and facilitate bacterial invasion [27,37].
4. Degenerative arthritis	Cartilage breakdown and synovial damage create irregular surfaces that facilitate biofilm attachment [1].
5. Low-grade inflammation	Chronic inflammatory state alters synovial fluid composition, impairing local immune defenses [37].
6. Hypoxic environment	CPP crystals contribute to low oxygen tension in the joint space which promotes anaerobic bacterial growth and reduces immune cell function [41].
7. Acidic environment	CPP crystals contribute to decreasing the pH in joint spaces which impairs neutrophil activity and antibiotic efficacy, favoring bacterial survival [41].

This report highlights the risk (and presumed variability) of non-traditional therapies, particularly ozone/prolozone therapy, due to its non-standardized composition. Not all homeopathic/alternative medicine interventions are held to rigorous standards of care backed by scientific evidence, and there are currently no established guidelines on the production, procurement, or sterility of such injectables. Although the cause of infection cannot be ascribed to any single factor, the overall pattern, including repeated injections, undocumented technique and procedural details, continued treatment despite worsening symptoms, and a potentially immunocompromised state, together represents a particularly instructive aspect of this report. This case underscores the importance of strict regulation of the production, sterility, and administration of these products.

*C. acnes* microbiology and pathophysiology of orthopaedic infection

*C. acnes* is an aerotolerant, anaerobic, Gram-positive bacillus commonly colonizing skin and pilosebaceous units, particularly of the shoulder, neck, chest, and back [42]. Although it is best known for its role in acne vulgaris, where it generally demonstrates low virulence [31,32], *C. acnes* can cause chronic, insidious infection [43,44]. This is primarily attributed to biofilm formation, which facilitates colony establishment and evasion from the immune system [22,31,45]. Virulence and inflammatory potential can vary across *C. acnes* strains, contributing to differences in pathogenicity [31,32]. The organism can also persist in a dormant state within macrophages for up to eight months *in vitro* [46]. Due to these biological characteristics and slow growth, *C. acnes* infection can be difficult to detect and eradicate [22,47,48]. Consequently, *C. acnes* requires a longer incubation period than other common bacteria [42]. Accordingly, cultures of fluid or tissue should be held for at least 14 days [49-51], as was done in this case.

Because *C. acnes* is a common skin commensal and potential contaminant, obtaining three to six deep-tissue samples is recommended to increase diagnostic specificity [52,53], and two or more separate positive cultures are considered indicative of true infection rather than contamination [28,30,54,55]. However, these criteria are not absolute, as multiple positive cultures for *C. acnes* do not always confirm infection [56-58]. In our patient’s case, two positive deep-tissue cultures, in conjunction with her signs, symptoms, and intraoperative findings, supported the diagnosis of septic arthritis.

Bacterial septic arthritis in association with ozone/prolozone or corticosteroid injections

The hip is the most common site of bacterial septic arthritis of the native joint [59,60], whereas involvement of the native shoulder joint is relatively rare [61]. *Staphylococcus aureus* (*S. aureus*) is the causative organism for the majority of these cases [62]. Other frequently isolated organisms include various *Streptococcus* species, Gram-negative bacilli, and *Neisseria gonorrhoeae*, with *C. acnes* and other anaerobic bacteria being rare in this setting [62,63]. Notably, *C. acnes* is the most common organism associated with suspected postoperative shoulder infections [42,46,64-67].

For the purpose of this review, septic arthritis of the native shoulder joint includes infections occurring in joints without surgery/prosthetic material. Thus, it encompasses both spontaneous cases and those temporally associated with intra-articular procedures such as injections or aspirations (PASA). We located only three reported cases of *C. acnes* involving the native (non-prosthetic) shoulder joint [55,68] (Table 3), and approximately 25 cases involving other large native joints [55,69-77]. Only one of these [72] involved concurrent crystal arthropathy (gout). Furthermore, we located only three described cases of bacterial septic arthritis of native joints following therapeutic ozone injections [78-80], none of which were caused by *C. acnes* (Table 4).

**Table 3:** Cases of C. acnes involving the native (non-prosthetic/non-surgical) shoulder joint Native shoulder joint infections were defined as infections occurring in shoulders without prior surgery or implanted hardware; our case, which included prior injections and aspirations, was included. * Age in years, M = male, F = female, CPPD = calcium pyrophosphate deposition disease, RA = rheumatoid arthritis

Author	Age, Sex*	Injection preceding? Substance?	Risk Factors
Keys et al. (2022) [55]	35, F	No	Sickle cell disease, avascular necrosis
Sulkowski et al. (1994) [81]	71, M	No	Osteoarthritis
Delyle et al. (2000) [82]	17, F	No	Arthritis, facial acne vulgaris, HLA-B27+
Current Case	58, F	Multiple ozone/prolozone injections, corticosteroid injections	CPPD, seronegative RA, and osteoarthritis

**Table 4:** Cases of infections in large joints following ozone/prolozone injections * Age in years, M = male, F = female, CPPD = calcium pyrophosphate deposition disease, RA = rheumatoid arthritis

Author	Age, Sex	Joint	Injection details	Organism	Notes
Seyman et al. (2012) [78]	83, M	Knee	Intra-articular ozone injections	Pseudomonas aeruginosa	Symptoms began four days after ozone injections
Yassin et al. (2022) [80]	not stated	Knee	One weekly intra-articular ozone injection for three weeks	not stated	Prior quiescent chronic osteomyelitis in knee region
Cortez et al. (2026) [79]	65, M	Likely acromioclavicular	One single session of ozone therapy 15 days prior to admittance to emergency department	Staphylococcus aureus	History of HIV, hypothyroidism, and hypertension
Current Case	58, F	Shoulder (glenohumeral)	Multiple ozone/prolozone injections, corticosteroid injections	Cutibacterium acnes	CPPD, seronegative RA, and osteoarthritis

Established risk factors for septic arthritis of native joints include advanced age, diabetes, illicit IV drug use, pre-existing inflammatory or degenerative joint conditions such as RA and osteoarthritis, and immunosuppression [62,83]. Crystal arthropathies, including pseudogout and gout, are also associated with an increased risk of septic arthritis, even in the absence of recent intra-articular corticosteroid injection [84-86]. Because the clinical presentation of septic arthritis overlaps significantly with that of crystal-induced arthropathy (e.g., joint swelling, redness, warmth, and severe pain), these conditions can be difficult to differentiate [86]. For this reason, Gram stain and synovial fluid culture testing are prudent even when crystals are identified [86,87], as was done with our patient.

Septic arthritis is a rare complication after intra-articular corticosteroid injections in large joints, with recent estimates of incidence ranging from 0.002% to 0.093% [88-90]. Additionally, Geirsson and colleagues reported a septic arthritis frequency of 0.14% after arthroscopy and 0.037% after arthrocentesis (joint aspiration) [91]. Although these estimates are generally reported per procedure rather than cumulatively across multiple injections, repeated intra-articular injections (typically corticosteroids) and aspirations represent a recognized risk factor for joint infection [24,36,92,93], particularly when sterile technique is inadequate (discussed further below) [94]. However, comparable epidemiologic data for intra-articular ozone/prolozone injections remain limited.

In the case described in this report, it is important to note that the patient underwent multiple aspirations and corticosteroid injections after the ozone/prolozone treatments, creating substantial confounding. Thus, attribution to any one treatment is speculative and cannot be distinguished from the risk associated with repeated invasive procedures in general, especially with some injections having unknown composition. This, along with the lack of early cultures and inconsistent sampling, limits our ability to draw conclusions about the source of the infection.

Do crystal arthropathies contribute to a deep joint environment that increases the risk of septic arthritis, similar to proliferative inflammatory diseases?

It is possible that gout and pseudogout are not merely clinical imitators of infection. Concomitant crystal arthritis (i.e., gout or pseudogout) with septic arthritis is rare, but cases have been documented. For example, in a 2007 retrospective study, Shah et al. [85] reported on all patients with synovial fluid crystals in aspirates from various large joints sent to their laboratory over a seven-year period. Of 265 aspirates containing monosodium urate (gout; n=183), calcium pyrophosphate (pseudogout; n=81), or both (n=1), only four (1.5%) had positive cultures indicating septic arthritis. The four concomitant cases involved one ankle, one hip, and two knee joints; one contained gout crystals, and three contained CPP crystals. Hence, three of the 81 samples (3.7%) containing CPP crystals had concomitant septic arthritis. Furthermore, a study by Lim et al. [84] demonstrated that gout is associated with a significantly increased risk of developing septic arthritis when compared with matched controls. Among 72,073 patients with gout and 358,342 without, those with gout were 2.6 times more likely to be diagnosed with septic arthritis. The authors noted that patients with gout may undergo more intra-articular injections compared to the general population, which may partially account for this elevated risk.

In this context, we pondered this question: Do CPP crystals contribute to creating a ‘dysbiotic’ joint microenvironment [1] that increases the likelihood that bacteria (via needle puncture or bacteremia) will settle into affected joint spaces and potentially lead to infection? Inflammatory diseases such as RA are associated with increased susceptibility to septic arthritis, potentially due to chronic synovial inflammation and sub-synovial neovascularization, which may impair host defenses and facilitate bacterial invasion [37]. This likely reflects the fact that synovial membranes generally have rich vascularity without a limiting basement membrane [39], and agents/diseases that increase production of extracellular matrix proteins (e.g., fibronectin-, fibrogen-, and laminin-binding proteins) contribute to creating an environment favorable for bacterial adhesion [40]. 

We considered whether the presence of CPP crystals similarly perturbed the synovial membrane of our patient’s shoulder. Additional possibilities include evidence that: (1) CPP crystals contribute to creating a hypoxic, acidic joint space environment that is favorable to anaerobic biofilm-forming bacteria [41], and (2) CPP crystals can trigger a strong immune response within the joint and surrounding tissues [95,96] (Table 2). Hence, pseudogout is not directly immunosuppressive; rather, CPP crystals activate the NLRP3 inflammasome, leading to robust neutrophilic synovitis and elevated inflammatory markers [97]. Notably, Abdullah and Kabil [98] have shown in a rat model that ozone therapy alleviates acute gouty arthritis through NLRP3 inflammasome inhibition. While these mechanisms, including hypoxia, acidosis, increased bacterial adhesion, and altered immune response, appear biologically plausible, they are not directly demonstrated in our patient and thus represent clinical hypotheses.

Routes of bacterial inoculation into a large synovial joint: multiple injections within the same joint space increase susceptibility to infection

The pathogenesis of PASA can be complex, with bacterial introduction into the joint occurring via various routes, including direct inoculation (e.g., percutaneous skin puncture), contiguous spread from nearby infection, or hematogenous dissemination (i.e., via bacteremia) from a distant location [99] (Figure 4). Hematogenous spread is more commonly seen with *S. aureus* and is considered unlikely with *C. acnes* [62,63,75,100]. Consequently, during management of our patient, we considered it extremely unlikely that the source of her *C. acnes* infection was hematogenous seeding (e.g., from a remote acne lesion or other occult source). This reflects the conventional understanding that *C. acnes* infections arise almost exclusively from contiguous spread or direct inoculation via needle puncture or surgical incision over the target joint [1,24,92,101]. However, there is emerging evidence that low-grade *C. acnes* bacteremia is possible and may seed anatomic locations where normal physiology or vascularity has been perturbed (i.e., a local ‘dysbiosis state’) (Figure 4). This possibility is suggested by findings of a controlled study in a rat model by Jia et al. [38], where *C. acnes* bacteremia was induced in the setting of a concomitant needle-puncture-induced intervertebral disc injury (which caused neovascularization of the disc periphery). This, along with the findings of two prior studies, supports the idea that: (1) human intervertebral disc colonization by *C. acnes* is thought to follow “chronic, low-grade bac­teremia” [102]; and (2) *C. acnes* may also be transported by macrophages that accumulate around annulus fibrosus (outer intervertebral disc tissue), which rup­tures following intervertebral disc herniation, subsequently releasing the bacteria in association with local neovascularization [103]. A similar explanation may be applicable to our patient, where repeated needle punctures could have caused microtrauma to the shoulder synovial membrane, thus promoting seeding of *C. acnes*.

Multiple preoperative corticosteroid injections in knee, shoulder, and hip joints have been associated with a higher likelihood of positive intraoperative bacterial cultures [24,36,92]. Evidence supporting this association was provided by Booker et al. [92], who investigated *C. acnes* prevalence in the subcutaneous fat and posterior shoulder joint capsule in patients undergoing surgery for frozen shoulder syndrome or glenohumeral instability. Five tissue samples were collected per patient. Skin preparation consisted of 2% chlorhexidine gluconate in 70% alcohol, followed by placement of exclusion drapes. Results showed a statistically significant (p<0.01) association between *C. acnes* growth in the posterior shoulder joint capsule and two factors: (1) a history of prior glenohumeral injection (including corticosteroid or contrast agent), and (2) colonization of *C. acnes* in the subcutaneous fat (grown in cultures obtained during surgery). Hence, the two preoperative diagnoses were not associated with *C. acnes* in the deep joint tissues. Additional support for the potential role of repeated injections in causing infection comes from a 17-year retrospective study by Larghi et al. [36], which showed that five of 11 patients (45%, four knees and one shoulder) who developed septic arthritis following joint injection had a history of multiple intra-articular injections. The injection agents associated with infection were corticosteroids (seven cases), hyaluronic acid (three cases), and platelet-rich plasma (one case). These findings suggest that the repeated intra-articular injections/aspirations performed in our patient’s shoulder are likely the strongest correlate of her infection. Furthermore, the most probable source of the *C. acnes* was her skin commensal population, though pathogenic transformation of commensals in her shoulder joint tissue cannot be excluded [101].

The possibility of commensal intra-articular *C.* *acnes* prior to injections/aspirations

While *C. acnes* is a common skin commensal, especially in sebaceous areas of the shoulder, the deep shoulder joint has traditionally been considered sterile. This assumption has recently been challenged by several studies suggesting that a native shoulder microbiome may exist. Qiu et al. [104] used 16S ribosomal RNA sequencing of total extracted DNA to determine whether bacterial commensal organisms exist in the tissue of the deep native shoulder joint. They detected genomic DNA from various *Acinetobacter* species and from the Oxalobacteraceae family in 74% of rotator cuff tendon samples. However, *C. acnes* DNA was detected only in the skin and not in the deep shoulder tissues, seemingly validating the idea that *C. acnes* infections are the result of skin contamination during surgery and not from the opportunistic expansion of resident (commensal) *C. acnes* in the shoulder joint. However, it later became apparent that their study was flawed because the polymerase chain reaction primers used were unable to detect most strains of *C. acnes* [105,106]. As a result, the apparent absence of *C. acnes* in deep shoulder joint tissues likely was a false-negative finding.

Later studies provided direct support that *C. acnes* can be a commensal within the native shoulder joint. Hudek et al. [107] detected *C. acnes* in deep shoulder tissues in 10 out of 23 (43.5%) patients with no previous injections (multiple specimens were obtained during first-time arthroscopic and open shoulder surgery). Additionally, a meta-analysis by Narulla et al. [1] evaluated culture data from 3,283 native shoulder joints and found microorganisms in 50.8% of cultured samples, with *C. acnes* representing the most common organism detected. They propose that in ‘dysbiosis states’ the shoulder microbiome is a potential source or contributor to common shoulder pathologies of unclear etiology, like osteoarthritis, rotator cuff tears, and adhesive capsulitis. In contrast, they argue that in ‘normobiosis states’ the shoulder microbiome is protective against pathology.

Our patient’s risk factors, such as seronegative RA, pseudogout and osteoarthritis, together with needle-puncture-induced microtrauma and exposure to injected substances, may have facilitated the pathogenic transformation of an otherwise commensal *C. acnes* population. The potential role of intra-articular ozone in this process is difficult to interpret, given its bactericidal properties, which would theoretically reduce the bacterial burden within the joint. However, these effects could have been rendered ineffective through dilution in synovial fluid, interaction with organic material, limited exposure time [108], or interference from components of the proprietary prolozone mixture used in her second, third, and fourth injections. Consequently, the assumption that an ozone injection is invariably protective against infection must be viewed cautiously.

Ozone/prolozone therapy in orthopaedics: limited evidence supporting clear efficacy

The safety and effectiveness of ozone injection therapy remain incompletely established, and comparative data are particularly limited for shoulder osteoarthritis and crystal arthropathies, including gout and pseudogout [109,110]. The FDA has stated that: “Ozone is a toxic gas with no known useful medical application in specific, adjunctive, or preventative therapy” [111]. Despite this, ozone and prolozone therapies have gained increasing attention as alternative treatments for various musculoskeletal conditions [5,9,108], especially as a replacement or complement to corticosteroid injections. For example, Buechel [9] reported symptomatic improvement after administering prolozone injections to 26 patients with diverse peri-articular inflammatory conditions in the proximity of partial and total shoulder and knee replacements, including hip bursitis and peri-articular knee tendonitis. However, interpretation of these findings is limited by small sample sizes, heterogeneity of treated conditions, and the absence of control groups.

Skin preparation to reduce *C. acnes* infection risk

Another important risk factor for post-injection septic arthritis includes poor sterile technique [93,94,112,113]. Even with proper sterile technique, conventional antiseptic preparations may not sufficiently penetrate the deeper dermal and hypodermal layers where *C. acnes* resides [114-116]. Supporting this limitation, a placebo-controlled trial demonstrated that *C. acnes* grew in culture in 70% of dermal biopsy specimens despite standard skin preparation with 2% chlorhexidine gluconate and 70% isopropyl alcohol [117]. 

Benzoyl peroxide has emerged as a promising adjunct for reducing deep *C. acnes* burden prior to shoulder procedures. Unlike standard antiseptics, benzoyl peroxide penetrates the deep sebaceous gland tissue in the dermis and epidermis, exerting bactericidal activity through oxidative free radical generation within pilosebaceous units [114-116,118,119]. In view of these studies, a pre-procedural at-home regimen of topical benzoyl peroxide application has been proposed to reduce deep follicular *C. acnes* colonization [115,116,118,119], followed by standard antiseptic preparation immediately prior to the procedure to reduce the surface-level microbial burden. This combined approach may offer more comprehensive antiseptic coverage and improve outcomes. Patients with major risk factors for infection could receive a short course of oral prophylactic antibiotics, which have been shown to significantly reduce the burden of *C. acnes* in the skin of the shoulder region [116]. Such an approach could be implemented within several hours of shoulder injections in appropriately selected patients. This hypothesis requires further investigation, and additional studies of such antibiotic prophylaxis and suggested skin preparation measures are needed.

## Conclusions

We report a rare case of *C. acnes* PASA of the shoulder in a patient with no prior surgery, pre-existing pseudogout, and a retrospective diagnosis of seronegative RA. The infection was associated with a series of eight percutaneous needle punctures for therapeutic injections and/or aspirations over a six-month period. This case highlights the cumulative procedural risk of repeated needle access to a single joint, the importance of sterile technique, and the diagnostic challenges of recognizing an indolent *C. acnes* infection amid coexisting pseudogout, osteoarthritis, and a rotator cuff tear. Despite consideration of various etiologies, the precise mechanism and causative route of infection remain uncertain. Direct inoculation from her commensal skin *C. acnes* population via repeated needle punctures seems most likely, though activation of a native shoulder joint commensal or failure to grow another organism (e.g., coagulase-negative *Staphylococcus*) cannot be ruled out. Clinicians should maintain a high index of suspicion for septic arthritis in patients undergoing repeated joint injections or aspirations, particularly when effusions fail to improve. Early cultures with prolonged incubation times should be considered when infection is in the differential, especially when injections include unknown ingredients or may have been administered with inadequate skin preparation. Clinicians should also recognize that the native shoulder joint may harbor *C. acnes* in some patients; however, there are currently no widely available methods for identifying such patients pre-procedurally. For patients with significant risk factors for developing post-injection septic arthritis in synovial joints, potential measures to reduce this complication include a two-step skin preparation regimen and the possibility of pre-procedure oral antibiotics, though these prophylactic strategies remain speculative. Further research is needed to establish evidence-based protocols for infection prevention in this setting.
